# The combination of vaccines and adjuvants to prevent the occurrence of high incidence of infectious diseases in bovine

**DOI:** 10.3389/fvets.2023.1243835

**Published:** 2023-10-11

**Authors:** Yiyang Yao, Zhipeng Zhang, Zhangping Yang

**Affiliations:** ^1^College of Animal Science and Technology, Yangzhou University, Yangzhou, China; ^2^Joint International Research Laboratory of Agriculture and Agri-Product Safety, Ministry of Education, Yangzhou University, Yangzhou, China

**Keywords:** cattle, vaccine, infectious disease, bovine immunity, adjuvant

## Abstract

As the global population grows, the demand for beef and dairy products is also increasing. The cattle industry is facing tremendous pressures and challenges. The expanding cattle industry has led to an increased risk of disease in cattle. These diseases not only cause economic losses but also pose threats to public health and safety. Hence, ensuring the health of cattle is crucial. Vaccination is one of the most economical and effective methods of preventing bovine infectious diseases. However, there are fewer comprehensive reviews of bovine vaccines available. In addition, the variable nature of bovine infectious diseases will result in weakened or even ineffective immune protection from existing vaccines. This shows that it is crucial to improve overall awareness of bovine vaccines. Adjuvants, which are crucial constituents of vaccines, have a significant role in enhancing vaccine response. This review aims to present the latest advances in bovine vaccines mainly including types of bovine vaccines, current status of development of commonly used vaccines, and vaccine adjuvants. In addition, this review highlights the main challenges and outstanding problems of bovine vaccines and adjuvants in the field of research and applications. This review provides a theoretical and practical basis for the eradication of global bovine infectious diseases.

## 1. Introduction

Cattle provide humans with large quantities of meat and dairy products and are an important source of protein. The growing demand for dairy products and beef has accelerated the growth of the cattle industry. Currently, the cattle industry has made remarkable achievements, however, outbreaks of infectious diseases have seriously hampered the growth of the cattle industry. These diseases not only affect the welfare and health of cattle but also cause huge economic losses. For instance, the annual impact of foot-and-mouth disease (FMD) in terms of visible production losses and vaccination in endemic regions alone amounts to between US$6.5 and 21 billion (1). Furthermore, the economic cost of *Mycobacterium tuberculosis* and mastitis to the global cattle industry is approximately $35 billion (2, 3). Brucellosis caused a median loss of $300 million to the Indian livestock industry with an average loss of $18.2 per buffalo (4). Additionally, other infectious diseases such as bovine viral diarrhea (BVD) (5), infectious bovine rhinotracheitis (IBR) (6), bovine lumpy skin disease (7, 8), and bovine leukemia (9) have caused varying degrees of losses to the cattle industry. These alarming figures highlight the urgent need to address and combat bovine infectious diseases. For example, the development and use of novel vaccines can mitigate the adverse effects of these diseases, safeguard animal welfare, and minimize economic damage.

**Figure 1:**
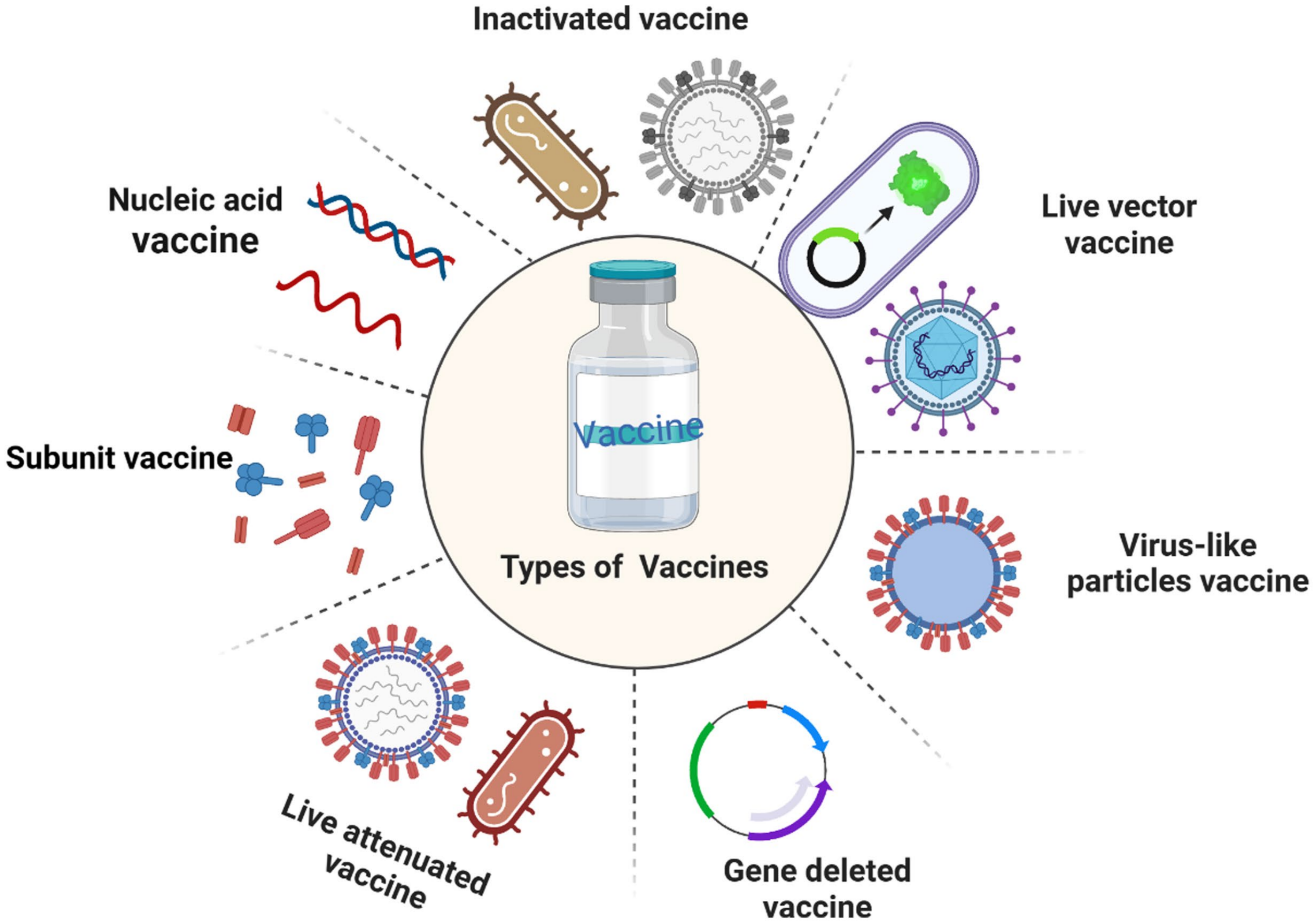
Common types of vaccines.

While antibiotics are effective in treating some bovine infectious diseases, concerns have been raised about the residual nature of antibiotics in animal products and the emergence of antibiotic-resistant bacteria. These problems not only affect the health and welfare of dairy cattle but also threaten human public health. As an important tool for effectively preventing bovine infectious diseases, vaccines play an indispensable role in preventing the re-invasion of pathogens. Vaccines can induce cellular and humoral immune responses. Currently, there are several types of vaccines, vaccines can be categorized according to the preparation method, which includes traditional vaccines like inactivated and live attenuated vaccines, as well as genetically engineered vaccines such as subunit vaccines, DNA vaccines, live vector vaccines, virus-like particles vaccines, and gene deleted vaccines (Figure 1).

Although traditional vaccines are widely used in bovine vaccine development and clinical application due to their high safety profile and easy preparation, they still have their limitations. For example, inactivated vaccines have a short immunization period and require multiple injections and strict storage conditions. In addition, antigens in live attenuated vaccines can persist in the host for a long time, potentially causing vaccinated animals to carry the virus for a long time. In addition, due to the constant mutation and evolution of the virus, traditional vaccines may not provide good immune protection. It can be seen that safe and immunogenic vaccines are essential for cattle health. The rapid development of genetic engineering technology has promoted the development of bovine vaccines. In addition, as an important component of vaccines, adjuvants play an important role in enhancing the response mechanism and efficacy of vaccines. In conclusion, a comprehensive understanding of bovine vaccines and adjuvants is essential for the practice management of pastures.

## 2. Types of bovine vaccines

### 2.1. Inactivated vaccine

Inactivated vaccines are the use of physical or chemical methods to inactivate a virus or bacterium so that it loses its virulence while maintaining its antigenicity (10, 11). Physical inactivation methods include irradiation, high-temperature heating, and ultrasound (12–14). Chemical inactivation methods include formalin, hydrogen peroxide, binary ethylenimine (BEI), and β-propiolactone (15–17). Inactivated vaccines primarily induce a systemic immune response through the production of neutralizing antibodies (12). Inactivated vaccines are extensively utilized in the development of commercial cattle vaccines due to their short development timeframe and high safety profile. The bivalent vaccine for Chinese FMD serotypes O and A is a typical inactivated vaccine. The inactivated vaccine for bovine lumpy skin disease developed based on the Neethling strain induced high levels of specific antibodies from 7 days post-vaccination (dpv). This vaccine provides good immune protection to cattle, with a protection period of up to 1 year (18). A study has found that an inactivated bovine influenza (BEF) vaccine needs to be immunized at least three times to stimulate the production of high levels of specific antibodies that provide effective immune protection for cattle (19).

It is evident that while inactivated vaccines are safe and do not induce infections, their duration of immune protection is relatively shorter, necessitating multiple immunizations to achieve long-lasting immune protection. Future advances in the development of inactivated vaccines could focus on improving their efficacy through the application of suitable adjuvants and the production of multivalent vaccines.

### 2.2. Live attenuated vaccine

Live attenuated vaccines use chemical or physical mutagenesis, genetic engineering, or attenuated culture to cause non-local mutations in the causative agent or to “knock out” virulence related genes in the strain, thereby reducing virulence while maintaining immunogenicity (20, 21). The most widely used live attenuated vaccines include the S19 and RB51 vaccines against bovine brucellosis. These vaccines exhibit reduced antigen virulence and can elicit a durable immune response (22). The attenuated antigens in live attenuated vaccines remain capable of replication, allowing for the induction of long-lasting immunity even without booster vaccinations or the use of adjuvants. Studies have shown that the S19 vaccine can confer significant immune protection to calves throughout their productive lifespan (23).

Currently, live attenuated vaccines have some limitations because vaccinated animals carry the virus in their bodies for long periods and pregnant animals vaccinated with live attenuated vaccines have a potential risk of vertical transmission. They may cause fetal complications or result in the birth of persistently infected (PI) calves (24).

### 2.3. Subunit vaccine

Subunit vaccines are vaccines that encode pathogen antigens into recombinant expression vectors and express the gene products (recombinant peptides or recombinant proteins) using prokaryotic or eukaryotic expression systems (25). Subunit vaccines contain only a part of the pathogen and lack viral nucleic acids, making them highly safe and non-pathogenic (26). E2Fc and E2Ft are subunit vaccines for BVDV (27). Despite their high safety profile, subunit vaccines usually show lower immunogenicity compared to other types of vaccines and therefore require multiple doses or the use of adjuvants. For example, a combined subunit vaccine of bovine respiratory syncytial virus (BRSV) NP and *Histophilus somni* IbpA DR2 (Quil A) reduced clinical signs and pulmonary pathology in calves infected with the virus but did not prevent virus excretion (28). Melatonin (MT) as an adjuvant to BVDV subunit vaccines has been shown to enhance T-cell immune response and alleviate pathologic injury by suppressing inflammation (29).

While the immunogenicity of subunit vaccines has improved significantly with the addition of adjuvants and other improvements, these are often reflected in higher vaccine costs. Therefore, future development of subunit vaccines will need to balance their immunogenicity and cost-effectiveness.

### 2.4. Virus-like particles vaccine

Virus-like particle (VLP) vaccines are vaccines made by expressing one or more structural proteins in a heterologous host system, which are then assembled into virus-like particles (30, 31). VLP is structurally similar to natural viruses but usually does not contain parental pathogen genetic material. VLP can generate a strong immune response even without adjuvant (32). It is a promising candidate for development and a safe and efficient vaccine. The major expression systems currently used for bovine VLP vaccines include bacteria (33), yeast (34), baculovirus/insect cells (B/IC) (35), and mammalian cells (36). For example, the FMD VLP vaccine developed using the *Escherichia coli* (*E. coli*) system significantly increased levels of foot-and-mouth disease virus (FMDV)-specific antibodies, neutralizing antibodies, and IFN-γ in cattle. In addition, a single dose of VLP immunization completely protected these animals from the homologous FMDV challenge (33).

Despite the remarkable results of VLP vaccines in the development of vaccines against bovine infectious diseases, it is worth noting that, at present, VLP vaccine research is mainly confined to small animal models, and less research has been conducted on bovine. In addition, the complex development process and high production costs have hindered their large-scale development. This highlights the need for further extensive research and more time to fully realize the potential of VLP vaccines in cattle.

### 2.5. Live vector vaccine

Live vector vaccines use genetic engineering techniques to clone antigens that protect against pathogens into non-toxic bacteria or viruses and express these genes as they replicate in the host, thereby inducing a specific immune response in the body (37, 38). It mainly includes viral live-vector vaccines and bacterial live-vector vaccines. They can induce cellular and humoral immune responses and have the advantages of high immunogenicity, and high specificity. In addition, they have lower toxicity risks and production costs (39).

Adenovirus (40), rabies virus (41), Newcastle disease virus (42), bovine herpesvirus 1 (BoHV-1) (43), and novel influenza virus (44) have been widely used in the development of bovine virus vector vaccines. For instance, a novel influenza virus vector (Flu-BA) vaccine expressing *Brucella. abortus* ribosomal proteins L7/L12 or Omp16 have been shown to induce cross-protection against *B. melitensis* in pregnant heifers. This vaccine provides a level of protection comparable to the S19 vaccine (45). Probiotics are commonly used in the development of live-vector vaccines for cattle. For example, the brucellosis outer membrane protein (OMP) 19 *Lactobacillus casei* vector oral vaccine has been shown to elicit systemic and mucosal immune responses in mice (46).

Although live vector vaccines can express multiple pathogen antigenic genes simultaneously and prevent multiple diseases with a single injection. Despite diminished virulence, live vectors can still cause potential harm to host organisms. Therefore, careful selection of live carriers is essential to ensure the safety and efficacy of vaccines.

### 2.6. Nucleic acid vaccine

#### 2.6.1. DNA vaccine

The DNA vaccine is a plasmid containing a gene encoding an antigenic protein that is introduced into the host and induces an immune response in the host cell by the expression of the antigenic protein by the host cell (47, 48). DNA vaccines have been widely used in the development of animal vaccines because of their ease of preparation, high safety profile, and long-lasting immune responses. However, DNA vaccines also face some challenges, such as the need for an effective delivery system to activate a strong immune response for optimal vaccine efficacy (49). Live bacterial vectors (50), and nanoparticles (51) are considered promising delivery systems for DNA vaccines. For example, DNA vaccines against tuberculosis using *Lactococcus lactis* as a vector and carrying an antigen encoding Ag85A have been shown to promote the production of proinflammatory cytokines (IFN-γ, TNF-α, and IL-6) and to induce a helper T cell (Th1) response. In addition, it induces the production of IgG and sIgA antibodies (52). Although commercial DNA vaccines against bovine infectious diseases have not been reported, current development strategies rely primarily on small animal models. In large animal models, plasmid delivery and expression are limited, resulting in weak immunogenicity of the antigen. However, the addition of adjuvants can alleviate this problem. For example, IL-18 as an adjuvant for FMD DNA vaccines induced higher titers of IgG antibodies and neutralizing antibodies, as well as significant T cell proliferative responses (53).

DNA vaccines have greater stability and safety than other types of vaccines. However, it is worth noting that despite significant progress in small animal models, research on DNA vaccines in cattle has been relatively limited. Therefore, the need for a large number of trials to fully evaluate the clinical efficacy of DNA vaccines in cattle is essential.

#### 2.6.2. mRNA vaccine

The mRNA vaccine is a sequence of mRNA encoding a specific antigen that is inserted into the body and expressed in the host, thereby inducing an immune response. mRNA vaccines include non-amplified mRNA and self-amplified mRNA (54). Similar to DNA vaccines, mRNA vaccines require a delivery system. They activate the innate and adaptive immune systems, but their exact mechanism of action remains unclear (55). Compared to other vaccine types, mRNA vaccines have some potential advantages such as efficient, low-cost production, and rapid development. They are considered safe because RNA is not infectious and can be degraded by normal cellular processes without inducing cellular infection or insertional mutagenesis (54, 56). Because of this, mRNA vaccines have been widely developed for human COVID-19 vaccines (57), but less so for cattle and other animals. A recent study showed that although mice vaccinated with the mRNA FMDV vaccine exhibited significant FMDV-neutralizing antibody titers, the vaccine provided only partial protection after the viral challenge (58). One study conceptualized a novel lumpy skin disease virus (LSDV) mRNA vaccine using subtractive genomics and reverse vaccinology approaches. Simulation analysis showed that the vaccine is ideal and may induce a strong immune response without causing sensitization (59). However, the mRNA vaccine requires further validation in cattle.

As third-generation vaccines, mRNA vaccines have considerable potential for the development of vaccines for cattle and other animals. However, there are fewer research reports on bovine mRNA vaccines, and the future development of mRNA vaccines should utilize improved delivery materials and explore the mechanism of action of different types of mRNA vaccines to accelerate the development of bovine mRNA vaccines.

### 2.7. Gene deleted vaccine

Gene deleted vaccine is a vaccine produced by knocking out the virulence genes of a virulent strain using molecular biology and genetic engineering techniques, resulting in the loss of expression of the causative gene and preserving the immunogenicity of the strain. Presently, the research on gene deleted vaccines mainly focuses on pigs, specifically for African swine fever and pseudorabies vaccines (60, 61) and cattle (BoHV vaccines) (62). Among them, the BoHV-1 gene deleted vaccine has gained popularity in Europe because of its similarity to strongly virulent infections and its ability to induce a strong immune response. The BoHV-1 deleted glycoprotein E (gE) vaccine is highly immunogenic, induces humoral and cellular immune responses, and distinguishes between infected and vaccinated animals (63). In addition, cattle vaccinated with a *Mycobacterium bovis* mutant strain (MbΔmce2) deleted for the mce2 gene expressed IL-10 at significantly higher levels than the parental strain. Mutant strains produce higher levels of Th1 cytokines when stimulated *in vitro* (64).

Gene deleted vaccines exhibit a high level of safety and efficacy by targeting and eliminating virulence-associated genes in strong strains. Gene deletion vaccines are considered to be the most ideal vaccines because they induce a strong and long-lasting immune response similar to infection with potent strains and also induce mucosal immunity when administered locally.

## 3. Vaccination differences among countries with developed dairy cattle industries in the world

Vaccination plays a crucial role in effectively preventing diseases in cattle. However, it is worth noting that cattle vaccination varies considerably among countries around the world, which may be related to regional differences, levels of economic development, policies, and disease prevalence. In China, FMD vaccination is mandatory. Other vaccinations are based on local government policy and specific farm conditions. Conversely, some developed countries have successfully eradicated diseases such as brucellosis, FMD, and bovine tuberculosis. Generally, there are significant differences in the level of vaccination of cattle between developing and developed countries. Table 1 provides an overview of the main immunizations for cattle in some developing countries (China, India, Brazil) and developed countries (United States, Australia, New Zealand) (65–74).

**Table 1:** Vaccination of cattle in different countries.

Vaccine	China	India	Brazil	New Zealand	America	Australia
Foot-and-mouth disease	✓	✓				
*Brucella bovis*	✓	✓	✓			
Bovine viral diarrhea	✓	✓	✓	✓	✓	✓
Infectious bovine rhinotracheitis	✓	✓	✓	✓	✓	✓
Bovine lumpy skin disease	✓	✓				
Bovine epidemic fever	✓	✓	✓			✓

“✓” indicates that the vaccine is widely immunized in this country.

As shown in the table, most bovine infectious diseases have been effectively eradicated in developed countries. Although vaccination of cattle is widespread in developing countries, outbreaks of infectious diseases cause significant economic losses to the cattle industry. In addition to improved control management and vaccination, improvements in commercial vaccines and the development of new vaccines play a critical role in the global eradication of bovine infectious diseases.

## 4. The current status of research on major bovine vaccines

### 4.1. Mastitis vaccines

Mastitis is one of the most common and highly prevalent diseases in dairy cattle, causing significant economic losses to the cattle industry (75–77). It is mainly caused by localized inflammation of the udder tissue due to invasion by mammary tissue by pathogenic bacteria (77). The main pathogenic bacteria include *Staphylococcus aureus* (*S. aureus*), *E. coli*, and *Streptococcus* (78–80). In addition, *Klebsiella pneumonia* (81) and *Burkholderia* (82) also cause dairy cattle mastitis. Antibiotics are widely used to treat mastitis in dairy cattle to reduce economic losses. However, some problems are gradually exposed, such as antibiotic residues in milk and bacterial resistance to antibiotics (83, 84). The dairy cattle mastitis vaccine could alleviate these problems. Currently, commercially available dairy cattle mastitis vaccines include the Lysigin^™^ vaccine against *S. aureus* (85), the J5 vaccine against *E. coli* (86), the Startvac^®^ vaccine (87), and so on. Furthermore, several experimental vaccines and group-specific autologous vaccines have been developed with remarkable success.

The current development of mastitis vaccines for dairy cattle is focused on *E. coli* and *S. aureus*. It has been demonstrated that vaccines of *Staphylococcus aureus* surface protein (SASP) and chromogranin surface protein (SCSP) significantly reduce the number of pathogenic bacteria in milk. Particularly, the SCSP vaccine has shown cross-protection against *S. aureus* mastitis in vaccinated dairy cattle (88). Additionally, the bovine mastitis *S. aureus*-cholera toxin A2/B vaccine has been found to stimulate the production of antigen-specific IgG and enhance the expression of CD4^+^ T cells and IL-4 in cattle (89). The Keyhole Limpet Hemocyanin-Enterobactin (KLH-Ent) vaccine has successfully elicited strong immune responses specific to Ent in dairy cattle while maintaining the diversity and health of gut microbiota (90). Furthermore, the *Streptococcus uberis* (*S. uberis*) mastitis vaccine has been shown to significantly reduce clinical signs of mastitis, bacterial counts in milk, and daily milk yield losses (91). When administered before calving, the *Klebsiella pneumoniae* glycoside receptor protein vaccine has effectively decreased the risk of both Klebsiella and total coliform mastitis by 76.9% and 47.5% respectively, while increasing milk yield by an average of 1.74 kg/day and reducing somatic cell count by 64.8% (92). The route of vaccination plays a significant role in the response of dairy cattle to mastitis vaccines. Local immunization has been shown to induce immunity associated with tissue-resident CD4 and CD8 memory T cells, as well as Th17 cell immunity (93). While some experimental and commercial vaccines have demonstrated effectiveness in preventing mastitis in dairy cattle, studies have found that vaccination with a commercial vaccine (Startvac^®^) resulted in a significant decrease in the 305 days milk yield and no significant difference in the incidence or duration of clinical mastitis cases compared to control groups (94–96). Additionally, the immune protection efficacy of the *E. coli* J5 vaccine tends to decrease during peak lactation in dairy cattle (97). Another study revealed that vaccination against enterotoxigenic *Escherichia coli* (ETEC) reduced death or culling due to mastitis, but did not have a preventive effect on the development of mastitis (98).

In conclusion, the current commercial mastitis vaccines have shown some success, but there are noticeable limitations. For example, mastitis is caused by a wide range of bacterial pathogens, and existing vaccines target only one or two specific causative organisms, which would result in a less effective vaccine (99). Furthermore, the distribution of mastitis-causing bacteria is regional. Epidemiologic surveys are essential to understand their distribution patterns. To effectively prevent dairy cattle mastitis, future vaccine research needs to be integrated with knowledge of immunology and vaccinology to fully understand the mechanisms by which mammary tissue responds to vaccines. Additionally, understanding local immunity guides determining the most appropriate immunization pathway for vaccines (3, 100). Novel adjuvants play a crucial role in enhancing the vaccine response. In addition, routine cleaning and sterilization of the nipple is an important part of preventing the development of mastitis. Therefore, a comprehensive understanding of the mechanisms of the role of the mammary immune system in innate and adaptive immunity is essential for the development of mastitis vaccines.

### 4.2. Foot and mouth disease vaccines

Foot-and-mouth disease (FMD) is a highly contagious and devastating disease that causes significant economic losses to the global cattle industry. For example, the cost of the FMD caused economic loss per cattle head in Ethiopia was approximately $76, while the impact of the 2001 FMD outbreak on United Kingdom agriculture and the food chain was approximately £300 million (101, 102). It is caused by the FMDV, a small RNA virus that primarily affects cloven-hoofed animals (103). Common symptoms of FMD infection in dairy cattle include fever, reduced appetite, lameness, blister formation on the mouth, feet, nose, and teats, as well as decreased milk production and abortion (104). There are seven FMDV serotypes: O, A, C, Asia l, SAT l, SAT 2, and SAT 3, of which serotype C is thought to be extinct (105, 106). It has been successfully eradicated in most parts of North America, Oceania, Europe, and South America through vaccination, culling, and strict management. However, FMD is still endemic in parts of Asia, Africa, and Latin America (107). The lack of cross-protection between serotypes of FMDV makes the development of new vaccines essential for the global eradication of FMD (108).

Currently, commercially available FMD vaccines are mainly multivalent inactivated vaccines. For instance, Tian Kang^®^ is a bivalent inactivated vaccine targeting FMD types O and A, which is available in China. In addition to inactivated vaccines, other types of vaccines are under development and clinical trials. The FMD subunit vaccine (AdtA24) provides long term immune protection for cattle, with the longest period of immune protection achieved through transdermal injection, and it also induces high levels of antibody titers. The internal ribosomal entry site (IRES) of FMDV has been identified as a critical virulence determinant, which informs the development of live attenuated vaccines for FMD (109). Another study found that the IRES RNA enhanced immunity and antibody titers induced by FMD vaccination and that IRES transcripts promoted early antibody responses in vaccinated mice (110). In addition, the *E. coli* vector FMD VLP vaccine induced strong and long-lasting humoral immune protection in cattle lasting about 6 months (111). Failure is an inevitable aspect of vaccine development. In a study evaluating FMD DNA vaccines (O1P1-3C minicircle, pTarget O1P1-3C, and mpTarget O1P1-3CLT plasmids), none of the DNA vaccines provided effective immune protection for cattle when administered alone (112). Another study of a synthetic peptide vaccine against FMD type A showed that the synthetic peptide vaccine provided lower immune protection and virus-neutralizing antibody titers to cattle after viral challenge compared to an inactivated vaccine (113, 114). This shows that DNA and synthetic peptide vaccines are less effective in cattle and need further improvement.

Although FMD inactivated vaccines have played an important role in controlling FMD outbreaks, there is still a need to develop highly effective and safe FMD vaccines with a long period of immune protection to achieve global eradication of FMD. Currently, FMD inactivated vaccine remains the most cost-effective and efficient tool in FMD endemic countries. The shortcomings of commercial vaccines are also evident, and the efficacy of existing vaccines can be improved by using novel adjuvants or stimulating powerful memory T cells (115). The serotype diversity and viral crossover of FMDV pose great difficulties for the global eradication of FMD. Strain-matching and epidemiological investigations in FMD endemic areas are important measures to understand the distribution of FMD serotypes (116). Future development of FMD vaccine can improve vaccine efficacy by expressing the empty capsid of FMDV using replication-deficient viral vectors, as well as improving and developing fully attenuated strains and applying novel adjuvants.

### 4.3. Infectious bovine rhinotracheitis vaccines

Infectious bovine rhinotracheitis (IBR) is a highly contagious disease caused by bovine herpesvirus type 1 (BoHV-1) (117). The BoHV-1 has three subtypes: BoHV-1.1, BoHV-1.2a, and BoHV-1.2b (118). BoHV-1.2a and BoHV-1.2b cause IBR, infectious pustular vulvovaginitis (118). The IBR disease outbreak has caused significant economic losses to the cattle industry (6). Vaccination and culling of seropositive animals have successfully eradicated BoHV-1 in Austria, Germany, Denmark, Finland, Sweden, and the Czech Republic (119). Currently, commercially available vaccines for BoHV-1 include modified live virus (MLV) vaccines, inactivated vaccines, gene deleted marker vaccines, and multivalent vaccines (which provide protection against BoHV-1, IBR, BVDV, Bovine parainfluenza 3, and other related viruses). An evaluation of eight commercially available IBR vaccines (including inactivated, modified weak, subunit, and gene deleted vaccines) demonstrated that the modified live vaccine and gene deleted vaccine was more effective. However, the subunit vaccine did not offer complete clinical protection, and there was a risk of calves becoming carriers of the virus after vaccination (120).

Additionally, several candidate vaccines have shown effectiveness in animal models. For instance, a triple-mutated BoHV-1 vaccine is significantly superior to the gE-deleted vaccine in terms of the number and duration of nasal virus shedding, virus-neutralizing antibodies, and cellular immune responses in calves after the viral challenge (121). Another study demonstrated that a triple-deleted (gG-/tk-/gE) BoHV-1 vaccine, which increased IgA levels in the serum of vaccinated calves, resulted in a significantly shorter duration of viral shedding after the viral challenge (122). DNA vaccine encoding BoHV-1 glycoprotein gD induces higher neutralizing antibody titers in calves than vaccines encoding BoHV-1 gC. In addition, DNA vaccines encoding gD elicit a higher immune response in calves and a significant 10-fold reduction in viral release (123). Additionally, the BoHV-1/5 gE vaccine expressed in Bilbao yeast effectively differentiates between vaccinated and infected cattle (124). One study evaluated the efficacy of four DNA vaccines against BoHV-1 in calves. The findings revealed that after 90 days of the initial vaccination, all calves were challenged with BoHV-1, resulting in severe IBR infection in every calve (125). Another study highlighted that the BoHV-1 DNA vaccine, encoding the gC and VP8 antigens, did not provide virologic protection compared to control groups, indicating the limitations of DNA vaccines in large animals and their reduced potency (123).

Currently, commercial vaccines play a crucial role in preventing IBR, but they do not offer protection against the effects of BoHV-1-induced latency in cattle. Genetic marker vaccines are highly desirable as they can effectively differentiate between vaccinated and infected animals. Regular serotype diagnosis is also essential for the eradication of IBR (126). In future vaccine development, it is important to consider the induction of strong and long-lasting immune protection through memory T cells, as well as the utilization of new adjuvants.

### 4.4. Bovine brucellosis vaccines

*Brucella bovis* is one of the common zoonotic diseases (66). This disease leads to a decrease in milk production, abortions, stillbirths, and reproductive issues in pregnant dairy cattle, while also affecting human reproductive capacity (127, 128). Although *Brucella* has been eradicated from livestock in developed countries such as Europe, North America, and Oceania, it remains prevalent in Latin America, parts of Africa, Asia, the Mediterranean region, the Middle East, and parts of South America (69, 129). Consequently, bovine brucellosis causes significant economic losses in endemic areas (4, 130).

Currently, the most commonly used vaccines against bovine brucellosis are the S19 strain weakened vaccine and the RB51 strain attenuated vaccine (131). S19 provides a higher level of protection (132). The RB51 vaccine is relatively safe and can distinguish between infected and vaccinated animals (133). However, both vaccines have limitations. The S19 strain interferes with serological tests and carries partial virulence, and the RB51 strain is resistant to rifampicin, which can lead to human infections (134). Additionally, the 45/20 attenuated vaccine and SR82 attenuated vaccine can also prevent bovine brucellosis, though the availability of the 45/20 vaccine is limited due to various drawbacks. The SR82 vaccine, with a protective efficacy similar to S19, is used in a few countries such as the Russian Federation (135, 136).

The development of an ideal vaccine is crucial for the global eradication of *Brucella bovis*. The outer membrane protein (OMP) of *Brucella bovis* is a potential immunogenic antigen widely used in the development of the bovine *Brucella* subunit vaccine (137, 138). Influenza A virus vector vaccines expressing *Brucella bovis* L7/L12 or Omp16 proteins showed high levels of protection in pregnant heifers with efficacy comparable to commercial vaccines S19 or RB51 (139). This recombinant vaccine also induces a strong T-cell immune response in cattle and provides high protectiveness against *Brucella bovis* infection (45). Moreover, the htrA cycl double-deleted bovine *Brucella* mutants (PHE1) vaccine has demonstrated good protectiveness in pregnant heifers (140). A combined DNA vaccine has been shown to induce high levels of antigen-specific CD4^+^ and CD8^+^ T cell responses, resulting in significantly higher antigen-specific IgG titers in calves (141). However, some test results have not been satisfactory. It was found that although mice vaccinated with the recombinant WRL7/L12 vaccine produced specific antibodies, they failed to provide immune protection to mice after the *B. abortus* 2,308 strain attack (142). Similarly, the recombinant cattle pox virus carrying *B. abortus* 18 kDa OMP did not provide immune protection when challenged with *B. abortus* 2,308 in mice (143).

Vaccination is a crucial measure for the prevention and complete elimination of bovine brucellosis (144). Most current commercial vaccines are attenuated, which has played a crucial role in preventing bovine brucellosis. However, the disadvantage is also clear, attenuated vaccines can make vaccinated animal transmitters and carriers of the virus, therefore some countries do not allow immunization (145). Therefore, the development of new and safe vaccines is essential in eradicating bovine brucellosis. Several promising vaccine candidates, such as recombinant vaccines and viral vector vaccines, have demonstrated safety and the ability to induce strong cellular immunity in mouse models. However, there is limited research on these vaccines in large animal models, and further progress is necessary. In the future development of vaccines for humans and bovine *Brucella*, it is worth considering the induction of mucosal immunity as well.

### 4.5. Bovine viral diarrhea vaccines

Bovine viral diarrhea (BVD) is a globally prevalent disease in cattle caused by the bovine viral diarrhea virus (BVDV) (146), which causes significant economic losses to the cattle industry every year (5, 147). BVDV belongs to the *Pestivirus* genus in the *Flaviviridae* family and there are three serotypes BVDV-1, BVDV-2, and HoBi-like virus (148). BVDV-1 can be isolated into at least 21 subgenotypes (1a–1u), while BVDV-2 has been described as four subgenotypes (2a–2d) (148). BVDV can cause increased body temperature, gastrointestinal damage, diarrhea, and miscarriage in cattle. Additionally, if pregnant dairy cattle are infected with non-cytopathogenic BVDV, the calves become persistently infected (PI) and carry the virus throughout their lives (149). Some countries (Sweden, Finland, and Norway) have completely eradicated BVDV (70).

Commercially available BVDV vaccines mainly include inactivated, live attenuated, and polyvalent vaccines. However, a study evaluating three commercially available vaccines demonstrated that while these vaccines provided fetal protection in pregnant dairy cattle, viremia was still detected in some vaccinated cattle that subsequently gave birth to PI calves (150). This highlights the importance of implementing biosecurity measures and diagnostic monitoring in addition to vaccination to ensure effective BVDV control. Therefore, the development of new vaccines plays a critical role in the eradication of BVDV.

The E2 protein is the most abundant surface protein of BVDV and contains the major BVDV-neutralizing antigenic site. It has been extensively utilized in the development of BVDV vaccines to induce BVDV-neutralizing antibodies (151, 152). The novel MHC-II-targeted BVDV subunit vaccine has shown a rapid and sustained neutralizing antibody response in cattle compared to conventional vaccines (153). Modified chimeric vaccines for BVDV-1 and BVDV-2 were effective in differentiating between infected and vaccinated cattle. These vaccines provided good immune protection to vaccinated cattle and significantly reduced the extent and duration of viremia and nasal shedding during viral attacks (154). Moreover, the BVDV DNA vaccine administered intramuscularly through an electroporation system (TDS-IM) in calves induced BVDV-specific humoral and cell-mediated immune responses more efficiently, leading to almost complete prevention of clinical signs of the disease (155). The current experimental and commercial vaccines for BVDV have certain limitations. Several studies have indicated that inactivated BVDV vaccination of pregnant cows can result in persistent infection of the newborn calves, leading to immune protection failure (156). Vaccination with a DNA vaccine encoding the BVDV E2 glycoprotein has shown strong humoral and cellular responses in vaccinated calves. However, the virus was still detectable in the leukocytes of vaccinated calves, suggesting that the vaccine did not provide complete immune protection (157). Additionally, cattle vaccinated with the BVDV E2 naked DNA vaccine exhibited limited immune protection when challenged with the virus (158). It has been observed that pregnant heifers and cattle that have received multiple doses of inactivated vaccine containing BVDV-1a may not be fully protected against infection with other subtypes of BVDV, including their fetuses, which may become persistently infected calves (159).

Existing BVD vaccines have proven effective in preventing BVDV infections. However, global eradication of BVDV is challenged by the long-term persistence of the virus in animals and persistent infections. There are multiple genotypes and subtypes of BVDV, and new strains may arise over time. To address these challenges, future vaccine research should focus on the E2 gene to develop more effective vaccines. Accurately distinguishing between vaccinated animals and infected animals is also crucial. It is crucial to eliminate animals with persistent infection and take measures to reduce the risk of BVDV entering the population. In addition, serotype-specific diagnosis is an effective measure for differentiating persistently infected animals. A comprehensive understanding of the molecular pathogenesis of BVDV and the mechanisms of immune response is essential. The development of new vaccines and the use of adjuvants will expedite the global eradication of BVDV.

In addition, other types of bovine vaccine candidates have shown remarkable results in animal models but require extensive experimental validation in cattle (Table 2).

**Table 2:** The effectiveness of other types of bovine vaccines in animal models.

Vaccine name	Target antigen	Injection dose	Protective effectiveness	Test subjects	Reference
*Escherichia coli* proteoliposome vaccine	Proteoliposome	0.2 mL	Decreasing bacterial count and tissue damage	Mice	(160)
*Escherichia coli* waaF subunit vaccine	waaF	80 μg	Significantly increased serum concentrations of IgG IL-2, IL-4, and IFN-γ and fecal concentrations of sIgA	Mice	(161)
Recombinant FMD vaccine	LIΔactAplcB-vp1	0.1 mL	Induced high levels of specific IgG antibodies and IFN-γ, TNF-α, and IL-2	Mice	(162)
Recombinant FMD AKT-T7 vaccine	AKT-T7 VP1strain	0.25 mL	Induced high levels of IFN-γ levels in mice with little effect on IL-4	Mice	(163)
The recombinant multiple-epitope protein of BoHV-1	gD/gC/gB	100 μg	The immune protective effect is similar to that of the inactivated BVD-IBR vaccine.	Rabbit	(164)
BoHV-1 glycoprotein B subunit vaccine	pET-32a-gB	100 μg	Induced guinea pigs to produce high levels of anti-gB antibodies and virus-neutralizing antibodies and significantly reduced viral shedding and lung tissue damage following IBRV virus infection	Guinea pigs	(165)
Cu–Zn superoxide dismutase (SOD) brucellosis DNA vaccine	pcDNA-SOD	10 μg	Induced a Th1 type of immune response and a protective response	Mice	(166)
Encoding ribosomal protein L9 brucellosis DNA vaccine	pVaxL9	100 μg	Provided increased immunogenicity and protection and increased IFN-γ-producing CD4^+^ and CD8^+^ T cells	Mice	(167)
Recombinant brucellosis subunit vaccine	Omp16 or Omp19	10 μg	A similar degree of protection to attenuated *Brucella* vaccines (S19 and RB51).	Mice	(168)
BVDV E2 protein oral vaccine	pPG-E2 DC pep /LC W56	200 μL	Induced anti-BVDV mucosal, humoral, and cellular immune responses via oral immunization	Mice	(169)
BVDV subunit vaccine	APCH-tE2	0.2 μg	Induce BVDV-specific neutralizing antibodies	Guinea pigs	(170)
BVDV E2 protein vaccine	pMASIA-tPAΔE2	30 μg	Elicits strong humoral and cellular immune responses	Mice	(171)

## 5. Vaccine adjuvants

**Figure 2:**
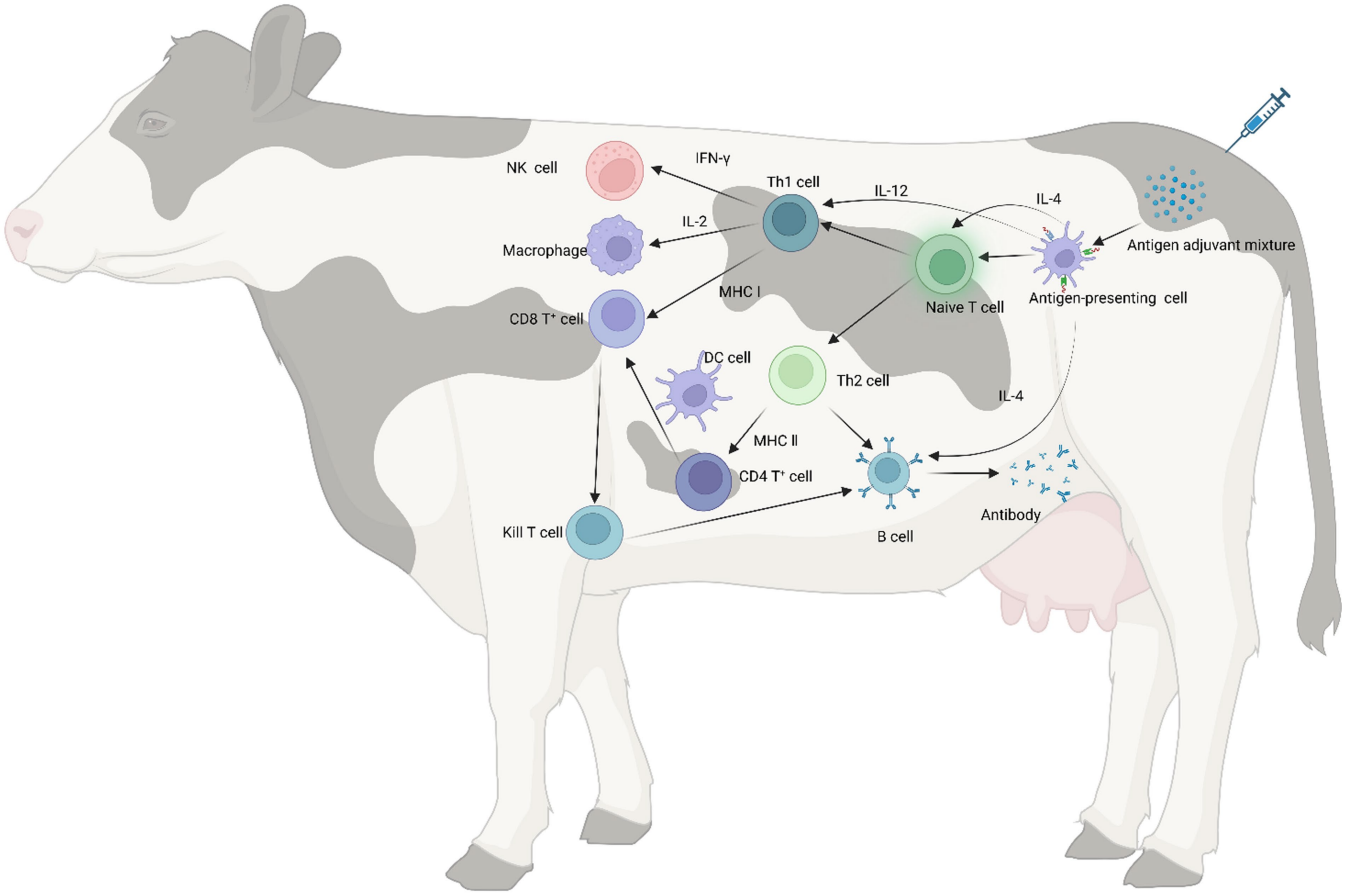
Immune mechanism of adjuvant. When an adjuvant is injected into the body along with the antigen, the adjuvant increases its surface area by adsorbing the antigen, thereby increasing the presentation capacity of antigen-presenting cells. The mixture of antigen and adjuvant stimulates naive T lymphocytes, causing them to differentiate into Th1 and Th2 cells. Among them, IL-4 secreted by APC can also promote the proliferation and differentiation of naive T lymphocytes. IFN-γ and IL-2 secreted by Th1 cells stimulate the proliferation of macrophages and NK cells and killer T cells, respectively, and in addition, antigenic peptide-major histocompatibility complex (MHC) activates CD8^+^ T cells to differentiate into killer T cells with the ability to specifically kill target cells, MHC II can promote CD4^+^ T cell maturation in synergy with dendritic cells (DC) to activate CD8^+^ T cells, and adjuvants enhance cellular immune responses through these pathways; IL-4 secreted by Th2 cells and naive T lymphocytes can assist in stimulating B cell proliferation and differentiation for antibody production. In addition, CD4^+^ T cells can activate CD8^+^ T cells through multiple pathways to differentiate into killer T cells, which can promote antibody production by B cells, thereby enhancing humoral immune responses.

**Figure 3:**
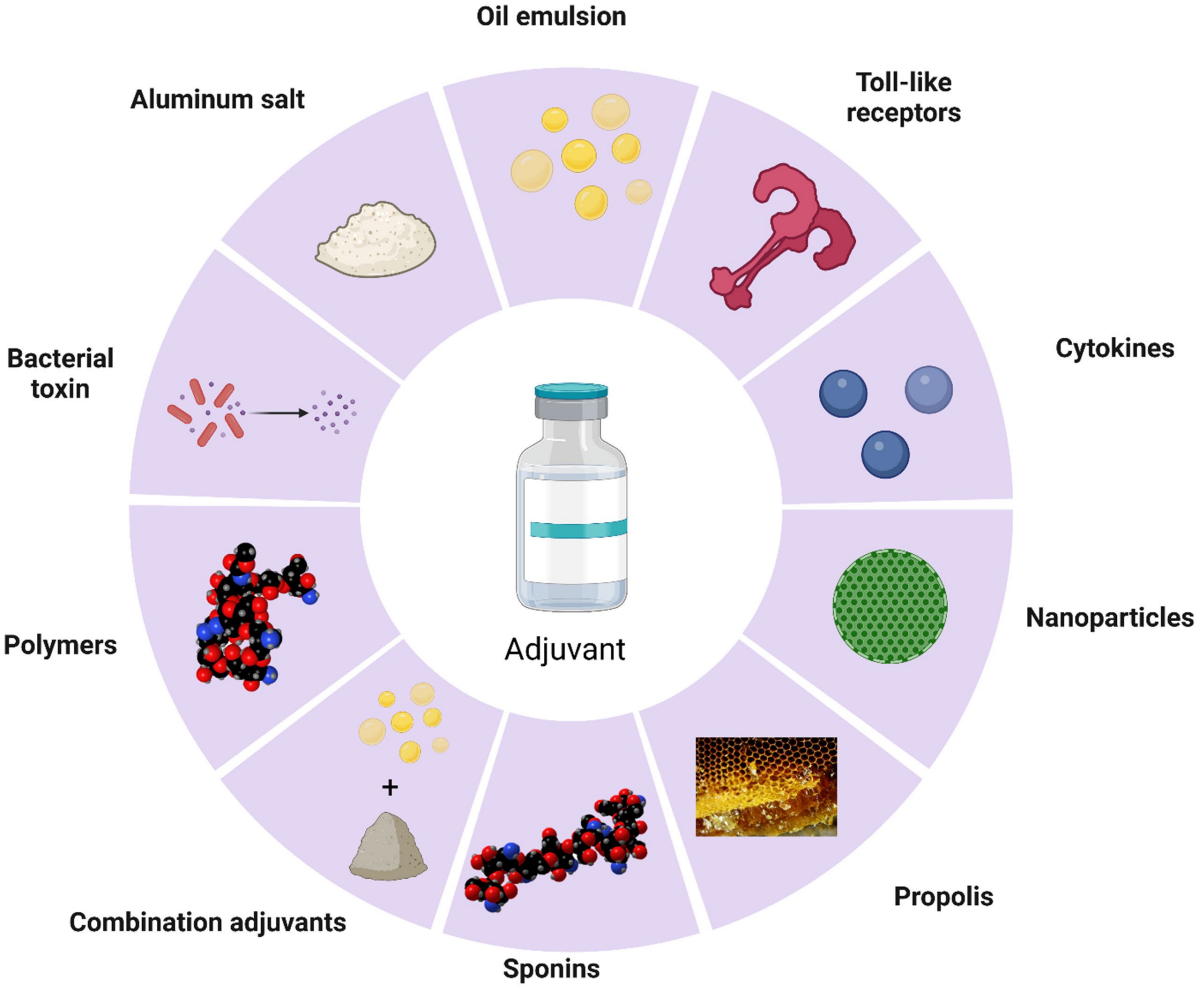
Types of cattle vaccine adjuvants.

Adjuvants, as important components of vaccines, not only increase the immunogenicity of the antigen but also improve the extent and durability of the immune response (172, 173). The current mechanisms of adjuvants in cattle remain unknown. However, broadly speaking, adjuvants can enhance cytokine secretion, promote dendritic cell maturation and antigen presentation, stimulate the proliferation and differentiation of T and B cells, and enhance the overall impact of vaccines (Figure 2). Adjuvants can be categorized broadly into antigen-presenting (aluminum salt, oil emulsions, nanoparticles) and immune-enhancing (toll-like receptors, cytokines, saponins, and propolis) (Figure 3).

### 5.1. Aluminum salts

Aluminum salt adjuvants mainly include aluminum hydroxide, aluminum phosphate, and alum. Aluminum adjuvants can induce strong humoral immunity in the body (174), induce differentiation of CD4 T cells into Th2 cells (175), and adsorb antigens, among other effects (176). Aluminum hydroxide adjuvant is the most commonly used adjuvant in commercial vaccines. Such as FMD vaccines (177), BVD vaccines (178), *Brucella* vaccines (179), and many others.

Aluminum salt adjuvants have several advantages, including their ability to induce strong and long-lasting humoral immunity and to promote macrophage response at the injection site (180, 181). However, they also have notable disadvantages, such as the small relative molecular mass of aluminum salt, leading to poor immunogenicity and weak induction of cellular immune response. Additionally, they may cause redness and allergic reactions at the injection site (182). The emergence of new vaccines, such as subunit vaccines and DNA vaccines, which do not rely on aluminum salts as adjuvants, has limited the use of aluminum salts. Currently, aluminum salts are not used independently in various cattle vaccine adjuvants but rather serve as a reference control in experimental vaccine candidates.

### 5.2. Oil emulsions

According to different dispersion states of oil emulsions, there are three types of oil-in-water (O/W) (183), water-in-oil (W/O) (184), and water-in-oil-in-water (W/O/W) emulsions. Among them, water-in-oil adjuvants are mainly used for veterinary vaccines. Oil emulsions adjuvant is the most commonly used adjuvant for FMD inactivated vaccines.

Currently, the main oil emulsion adjuvants used for application and research are MF-59 (185), ISA Montanide 25, and ISA 206 (186). MF59^®^ is an oil-in-water emulsion containing squalene, which induces a stronger humoral immune response than aluminum salt adjuvant. It tends to promote Th2 immune-biased responses and can enhance adaptive immunity by overcoming CD4 T cell trapping (187, 188).

Montanide ESSAI IMS D 12802 VG PR, as an adjuvant for the FMD vaccine, provides cattle with high levels of mucosal IgA antibodies and INF-γ, and 100% protection against viral challenge at 4 dpv and 7 dpv, where the level of INF-γ is approximately twice as high as that of the commercial vaccine (which contains oil emulsion adjuvant) (189). The new Montanide ISA 61 VG oil base as an adjuvant vaccine for the FMD vaccine induced cattle to produce higher levels of antibody titers and improved humoral responses by at least 19% compared to the adjuvant vaccine with alum (190). It has been found that Montanide ISA 206B as an adjuvant for the FMD vaccine elicits a higher SAT2 neutralizing antibody response and three times higher levels of systemic IFN-γ response observed at 14 and 28 dpv when compared to Quil-A and provides long-term immune protection for cattle (191). A study found that recombinant BoHV-5 gD (rgD5) (ISA50V2) significantly induced a high level of humoral immune response in cattle, with a five-fold increase in total IgG and a four-fold increase in BoHV-1 and -5 neutralizing antibody titers in cattle vaccinated with the iBoHV-5 plus rgD5 vaccine, compared to the alum adjuvant group (192).

Oil emulsion adjuvants protect antigens from degradation, prolong antigenic stimulation, and enhance immune response. However, it is important to note that continuous stimulation of oil emulsion adjuvants may cause tissue damage, stress reactions, and potential safety hazards associated with mineral oil residues (193). In addition, the size of the oil emulsion adjuvant droplets affects the effectiveness of the immune response. Therefore, it is critical to select the appropriate—size of emulsion droplets for a particular antigen to optimize the vaccine response.

### 5.3. Toll-like receptor agonists

Toll-like receptors (TLRs) play a critical role in innate immunity in mammals. These protein molecules are responsible for recognizing pathogen-associated molecular patterns (PAMPs) and initiating the immune response by releasing pro-inflammatory factors (194). TLRs are widely studied in humans for the treatment of cancer and some infectious diseases (195). However, the use of TLRs in cattle has been limited. Like humans, cattle also express TLR1–TLR10 (196). Because TLRs are specific, each TLR recognizes a different antigen.

TLR2 and TLR9, as adjuvants to recombinant Neospora caninum profilin (rNcPRO), induced cattle to produce higher levels of IFN-γand induced a prolonged recall B-cell response compared to aluminum hydroxide (197). The TLR4 agonist glucopyranosyl lipid A (GLA) and the TLR7/8 agonist assimilate (R848) were utilized as adjuvants for a candidate subunit vaccine (ID83 fusion protein). While these adjuvants induced the production of ID83-specific antibody IgG1 in cattle (198). TLR-7/8, as an adjuvant for the FMD vaccine, induces a strong humoral immune response and a long-lasting memory response in cattle, with antibody titers and virus-neutralizing antibody titers approximately twice as high as those of commercial adjuvants at 28 dpv, as compared to conventional adjuvants (aluminum hydroxide and saponin) (199).

It is worth noting that TLR agonists as vaccine adjuvants have not been extensively studied in cattle. The mechanisms of action for each TLR in this context remain unclear and require further investigation.

### 5.4. Cytokines

Cytokines (CK) are small soluble protein-like substances produced by cells of the body after stimulation by antigens (200). Cytokines that have immune adjuvant effects include interleukin (IL), interferon (IFN), granulocyte-macrophage colony-stimulating factor (GM-CSF), and chemokines. Among them, IL-2, IL-4, IL-6, IL-12, IL-17A, IFN-α, GM-CSF, and IL-18 are often used as adjuvants for cattle vaccine candidates.

For instance, the use of IFN-α and IL-2 as adjuvants in bovine infectious keratoconjunctivitis (IBK) vaccines has been shown to significantly enhance the protective efficacy of the vaccine and reduce ocular damage caused by *Moraxella bovis* in calves (201). Recombinant IL-17A as an adjuvant for the BoHV-5 subunit vaccine has been found to significantly increase virus-specific IgG and neutralizing antibody levels in cattle compared to other controls (202). IL-18 as an adjuvant for FMD DNA vaccine compared with the conventional inactivated vaccine containing Montanide ISA 2006, the IL-18 group was able to increase the levels of neutralizing antibodies and specific T cell proliferative responses and Th1 and Th2 cytokine responses in cattle. At 14 dpv, the level of IFN-γ produced was approximately double that of ISA 2006 (53). IL-6 as an adjuvant for BHV-1(gD) vaccine induced gDt-specific IgG and IgA antibody levels in calves, although it did not enhance the protective immune response to BHV-1 (gD) challenge (203).

Cytokines, as immunostimulatory adjuvants, exhibit diverse immunomodulatory functions and are highly biocompatible. They not only enhance specific immune responses but also improve nonspecific immunity (204). However, it is important to consider the susceptibility of cytokine activity to internal factors, hydrolytic enzymes, and other factors, as well as the relatively short half-life and high cost associated with their use as vaccine adjuvants.

### 5.5. Saponins

Saponins are natural products extracted from plants (205). Quila and QS-21 are commonly used as immune adjuvants. Quila stimulates the body’s Th1 immune response and also induces a CTL response (206). QS-21 enhances APC presentation and stimulates Th1 and Th2 cytokine secretion (207).

Saponin adjuvants have played an important role in the development of vaccines for BVD, IBK, and BRSV, as well as commercial FMD vaccines for cattle. For example, the addition of saponin to FMD oil emulsion vaccines significantly enhances the immune response in guinea pigs and cattle, inducing higher levels of neutralizing antibodies (208). ISCOM, as an adjuvant to the BRSV vaccine, was shown to reduce the replication of upper and lower respiratory viruses in calves and strongly stimulate lymphocyte production of gamma interferon (IFN-γ) (209). Similarly, the use of ISCOM as an adjuvant in the IBK vaccine has been found to significantly reduce clinical signs of IBK in calves and induce higher levels of specific antibodies (210). QS-21 as an adjuvant for FMD vaccine significantly increases early antibody levels in cattle (211). ISCOMATRIX^™^, as an adjuvant for *S. aureus* CP5 vaccine, induced heifers to produce higher levels of anti-bacterial and anti-CP5 IgG and IgG2 responses, which significantly increased the number of bacteria ingested by neutrophils at 7 days post-partum, in addition to the expression of IL-4, IL-10, IL-12, TNF-γ were all 1.25 times higher than that of the aluminum hydroxide group (212).

Saponins modulate innate immune cells and enhance non-specific immunity and humoral and cellular immunity (213, 214). However, they also have certain drawbacks such as hemolytic activity and weak toxicity (215). Future research on saponins could be conducted to facilitate the development of bovine vaccines by minimizing their toxicity and searching for novel saponin adjuvants.

### 5.6. Nanoparticles

Recently, significant progress has been made in the field of nanotechnology, particularly in the development of biodegradable nanoparticles for use as vaccine delivery systems. Nanoparticles can enhance humoral, cellular, and mucosal immune responses. In addition, they can store and release antigens. Therefore, they are considered very promising for vaccine delivery systems (216, 217).

Chitosan nanoparticles (CNP), silica nanoparticles, cationic solid lipid nanoparticles (cSLN), and polylactic acid-glycolic acid (PLGA) nanoparticles are used as vaccine carriers or adjuvants in the development of cattle vaccines. For example, recombinant cyclic nanoparticles used as an adjuvant in the bovine respiratory syncytial virus (BRSV) vaccine elicited the production of specific antibodies and specific cellular immunity in vaccinated calves, resulting in reduced clinical signs and viral loads compared to controls after BRSV challenge (218). Loading the sporozoite surface antigen p67C into silica capsules induced a robust T-cell immune response to the p67 antigen in cattle (219). In addition, encapsulation of bovine parainfluenza virus (BPI3V) vaccine with PLGA nanoparticles significantly increased the level of BPI3V-specific IgA antibodies in calf nasal mucus and was much higher than that of commercial vaccines (220). Chitosan-coated PLGA FMD DNA nanoparticle vaccine (Chi-PLGA-DNA), induced higher levels of mucosal, systemic, and cell immunity in cattle and produced approximately three times the level of sIgA of commercial vaccines. Although it does not provide complete clinical protection, it reduces disease severity and viral excretion and delays the onset of clinical signs (221).

Nanoparticles are efficient and safe as novel vaccine carriers, delivering antigens to immune cells and enhancing both humoral and cellular immune responses. In addition, nanoparticles can induce mucosal immunity, thus providing new ideas for vaccine development. Given these advantages, nanoparticles are highly desirable adjuvants and carriers in the field of vaccine development.

### 5.7. Combination adjuvants

The so-called combination adjuvant involves immunizing animals with two or more adjuvants combined with antigens (222). Although single adjuvants have achieved remarkable results in commercial and experimental vaccines, they have some drawbacks such as low immunization efficacy and insufficient protection. The problem can be solved by the use of complex adjuvants, which enhance cellular and humoral immune responses (223).

Currently, the main adjuvants used for cattle vaccine development include carbomer-saponin. CpG oligonucleotides, host defense peptides, polyphosphazene, saponin-oil emulsions, combined cytokine adjuvants (IL-1β IL-2), CpG ODN. Poly[bis (sodium carboxyethylphenoxy)]-phosphazene (PCEP), CpG ODN, or poly(I: C) combined with immune defense regulator (IDR) peptides have been used as adjuvants for the BVDV E2 vaccine, resulting in the induction of strong virus neutralizing antibodies and cell immune responses, including CD8 cytotoxic T cell (CTL) responses in cattle (224). Emulsigen and saponins as adjuvants for *Mycoplasma bovis* vaccine significantly induced humoral immune response and enhanced the production of specific antibodies, including IgA response, while also reducing lung lesions and providing good immune protection for calves (225). Furthermore, the combination of Mincle and STING stimulating ligands as adjuvants for the FMD vaccine has been found to induce high levels of antigen-specific and virus-neutralizing antibody titers during early vaccination and maintain long-lasting immune memory responses in cattle (199). The combination of CD40 ligand and Montanide^™^ GEL01 as an adjuvant to the BoHV-1 DNA vaccine increased serum neutralizing antibody levels compared to a single adjuvant and resulted in a significant decrease in bovine viral excretion and clinical scores following viral challenge, which was accompanied by a significant increase in the expression of IFN-γ and IL-4, which was approximately 2-fold greater than that of the single adjuvant group (226). A7 + ISA + CpG ODN RW03 as an adjuvant to the FMD vaccine induced a strong and long lasting anti-FMDV antibody response in cattle, and after viral challenge, the group provided effective immune protection to calves at a rate approximately 2 times that of the commercial vaccine. At day 84 of injection, the induced anti-FMDV antibody titer was approximately 2.1 times higher than that of the commercial vaccine (227).

Combination adjuvants not only address the limitations associated with single adjuvants but also exhibit enhanced immune responses, making them a promising area of research for vaccine adjuvants. Although some studies have shown promising results in large animal models, further field trials are necessary to validate their efficacy. Additionally, the cost implications of developing vaccines should be taken into consideration. Furthermore, the development of novel combination adjuvants specifically for vaccine candidates in dairy cattle is of utmost importance.

In addition to the above adjuvants, other novel adjuvants have achieved significant results in different animal models, but extensive field experiments are still needed to prove their efficacy and safety (Table 3).

**Table 3:** Effectiveness of novel candidate vaccine adjuvants in animal models.

Adjuvant name	Object	Effect	Reference
Astragalus polysaccharide (APS)	Pig	APS increased the phagocytic capacity of peritoneal macrophage, DC maturation, T-lymphocyte proliferation, expression of cytokines, and antibody production	(228)
*Chuanminshen violaceum* polysaccharides	Mice	Enhances both cellular and humoral immune responses	(229)
Purslane polysaccharides	Mice	Significantly enhanced the FMDV-specific cellular and humoral immune response	(230)
Green propolis ethanol extract	Cattle	Significantly increased the level of neutralizing antibodies in the BoHV-5 inactivated vaccine	(231)
Glycol chitosan	Cattle	Provides good immune protection	(232)
Polyphosphazene	Cattle	Enhanced the secretion of the cytokines IFN-α, TNF-α and IFN-γ *in vitro* and stimulates innate immune responses	(233)
Polyacrylic polymer	Cattle	Significantly increases the level of specific antigen IgA	(234)
Polyoxidonium	Guinea pigs	Stimulation of humoral and cellular immune responses to live brucellosis vaccines	(235)
Swine-derived *Lactobacillus acidophilus* SW1	Mice	Enhanced levels of FMDV-specific antibodies and FMDV-neutralizing antibodies significantly increased the secretion of IFN-γ and IFN-γ	(236)
Cholera toxin B	Mice	Significantly induced cellular and humoral immune responses	(237)
*Minthostachys verticillate* essential oil and limonene	Mice	Significantly increased the proportion of specific IgG (IgG1 or IgG2a) and CD4^+^ and CD8^+^ T cells producing IFN-γ	(238)
Boron	Mice	Reduced local inflammatory reactions induced by the Montanide adjuvants. Increasing the levels of anti-*S. aureus* antibodies	(239)
Cationic PLGA nano/microparticles	Guinea pigs	Induced high levels of antigen-specific serum IgG and IgA antibody responses and strong cell-mediated immune response	(240)
Silica vesicle nanovaccine	Mice	Induced stronger and higher memory responses, and stronger Th1 and Th2 responses	(241)

## 6. Concluding remarks

Currently, commercially available bovine vaccines mainly include inactivated, live attenuated, subunit and gene deleted vaccines. Diseases such as rinderpest, FMD, bovine brucellosis, bovine tuberculosis and BVDV have been successfully eradicated through vaccination in some countries. This shows that vaccination is an important measure for the prevention and eradication of infectious diseases. However, due to viral mutations and bacterial drug resistance, the development of new vaccines is crucial. While gene deleted vaccines and DNA vaccines have shown promising results compared to traditional vaccines, their safety and stability still need to be verified through extensive field trials. Additionally, adjuvants play a critical role in enhancing vaccine response. Efficient adjuvants can significantly improve vaccine efficacy, searching for novel adjuvants essential in the development of cattle vaccines. This review highlights the significance of various adjuvants, including aluminum salts, oil emulsions, saponins, cytokines, TLR, nanoparticles, and combination adjuvants, in vaccine candidates for dairy cattle. These novel adjuvants are considered ideal for enhancing vaccine response. However, due to limitations in sample size, further studies are required to confirm the safety and efficacy of these adjuvants.

The global eradication of bovine infectious diseases holds significant importance for the cattle industry and human health. However, currently commercial vaccines have certain limitations and fail to provide sufficient immune protection. The development of new vaccines often takes a lot of time and money. In the interim, modifying and enhancing existing vaccine formulations through the application of novel adjuvants, altering immunization routes, and increasing the number of immunizations, along with conducting extensive evaluation and optimization in large scale field trials, is crucial for effectively preventing existing diseases. The complete eradication of bovine infectious diseases is a collective global aspiration. A comprehensive understanding of the basics of the bovine immune system and the mechanisms by which different pathogenic bacteria infect the host is essential for the development of novel vaccines. In addition, combining knowledge of immunology and vaccinology to stimulate strong T cell immunity and utilizing novel adjuvants will accelerate the development of new vaccines.

## Author contributions

YY generated the initial draft. ZZ and ZY have revised the final content critically. All authors contributed to the article and approved the submitted version.
